# Nutrition and Aging

**DOI:** 10.3390/ijms24119265

**Published:** 2023-05-25

**Authors:** M. Hasan Mohajeri

**Affiliations:** Institute of Anatomy, University of Zurich, Winterthurerstrasse 190, 8057 Zürich, Switzerland; mhasan.mohajeri@uzh.ch; Tel.: +41-79-9381203

This Special Issue focuses on the importance of nutritional interventions for the delay of age-related conditions. Nutritional compounds have been shown to play a fundamental role in counteracting aging of the cardiovascular system, skeleton, brain, gastrointestinal tract, microbiome, and skin, among other organs in the human body. Recent scientific evidence documents the consequences of imbalanced nutritional habits and insufficient coverage with essential nutrients on the optimal development of the organs and maintenance of their functions throughout life. This Editorial will focus and comment on important topics discussed in individual reports included in this Special Issue.

A report by Bitar [1] studied the involvement of thrombospondin-CD47 signaling on the senescence of endothelial cells and angiogenesis in diabetes. Thrombospondin-1 (TSP1) is often secreted at sites of injury and tissue remodeling by many cell types, including fibroblasts and endothelial cells [2], playing an inhibitory role in angiogenesis. The anti-angiogenic effect of TSP1 is mediated by the binding of TSP1 to cell surface receptors, namely CD36 and CD47, with consequent suppression of key angiogenic markers, including nitric oxide (NO)/c-GMP-dependent signaling. The results presented here suggest that the overexpression of TSP1-CD47 signaling in diabetes may be associated with endothelial dysfunction, including impaired angiogenesis, cellular senescence, and a heightened state of oxidative stress. The author hypothesizes that TSP1-CD47 may have potential in the treatment of endothelial cell senescence in diabetes.

Microglial calcium signaling and its role in healthy brain aging was studied by del Moral et al. [3]. Using in vivo two-photon imaging, Ca^2+^ signaling and process extension of cortical microglia were determined in young adult (2–4-month-old), middle-aged (9–11-month-old), and old (18–21-month-old) mice. While the fraction of cells displaying spontaneous Ca^2+^ transients progressively increased with age, the frequencies and durations of the spontaneous Ca^2+^ transients followed a bell-shaped relationship, with the most frequent and largest Ca^2+^ transients seen in middle-aged mice. Moreover, in old mice, microglial processes extending toward an ATP source moved faster but in a more disorganized manner, compared to young adult mice. Altogether, these findings identified two distinct phenotypes of aging microglia: a reactive phenotype, abundantly present in middle-aged animals, and a dysfunctional/senescent phenotype, ubiquitous in old mice.

The function and health of microglia and astrocytes in the (aging) brain were the subjects of a review by Bok and colleagues [4]. Dietary restriction (DR) is shown to extend the lifespan in various organisms, but it is unknown whether DR has an antiaging effect in humans. Cumulative data suggest that proinflammatory activation of astrocytes and microglia is attenuated under DR. A major hallmark of aging is systemic, low-grade chronic inflammation throughout the body (termed inflammaging), as has also been shown to be associated with obesity and metabolic diseases. Chronic low-grade inflammation is a common feature of the aged brain [5] and can be deleterious in normal aging as well as in pathological aging related to neurodegenerative diseases. In this article, the available evidence for the anti-neuroinflammatory effect of DR, especially in aging and age-associated brain disorders, is provided.

In their comprehensive review, Gómez-Gómez and Zapico [6] aimed to describe the nutritional factors that may lead to the development of frailty, especially cognitive decline. Frailty was defined as a clinically detectable syndrome, related to the aging of multiple physiological systems, which prompts a situation of vulnerability. The etiology of frailty is multifactorial, and its pathophysiology is influenced by the interaction of numerous factors. Atherosclerosis, sarcopenia, cognitive deterioration, and malnutrition were suggested as major mechanisms triggering frailty. Malnutrition is associated with cognitive impairment, and an inadequate nutritional status predisposes one to cognitive frailty. Additionally, nutritional factors that may influence vascular risk factors will potentially affect dementia among patients with cognitive frailty. In this report, oxidative stress was identified as one important factor implicated in cognitive deterioration. Consequently, increasing antioxidants in the diet may be one therapeutic strategy for the management of these patients, due to a reduction in oxidative stress, either by their function as free radical scavengers or potentiating the antioxidant effect. On the other hand, the inappropriate use of antioxidants at high doses could have side effects and toxicity. Therefore, additional mechanistic studies are required as well as clinical trials to substantiate the clinical evidence of nutritional interventions to reduce brain aging.

The topic of dietary intervention to prevent the aging of skeletal muscle was the focus of the study by Receno et al. [7]. This study employed dietary curcumin supplementation, given to aged male F344xBN rats, which started at 32 months of age, a time at which muscle mass has already begun to decline. Aged rats exposed to prolonged dietary curcumin exhibited a greater muscle mass and produced more force in behavioral testing compared to control animals. Mechanistically, nuclear fraction levels of nuclear factor erythroid-2 related-factor-2 (Nrf2) were higher, and oxidative macromolecule damage was lower following the curcumin diet. Taken together, this study showed that curcumin may impart beneficial effects on aged skeletal muscle.

As the aggregation of amyloid-β (Aβ) peptides in the brain is one of the hallmarks of Alzheimer’s disease (AD), Shin et al. [8] tested whether inhibition of Aβ aggregation could be an effective strategy for AD treatment. The effects of Jowiseungchungtang (JWS), a traditional oriental herbal formulation, on Aβ aggregation, were investigated in an animal model expressing high levels of Aβ peptide in vivo. This study showed that JWS had inhibitory effects on Aβ aggregation, Aβ-induced pathologies, and improved adult hippocampal neurogenesis in the 5XFAD mice.

The microbiota residing in the human gut metabolizes dietary fibers and releases bacteria-produced metabolites, normally via fermentation. The (beneficial) effects of bacterial metabolites depend largely on consumed dietary fibers [9]. Saki et al. studied the impact of bacteria-produced hydrogen suppressing cellular senescence through scavenging cytoplasmic hydroxyl radicals (cyto·OH) [10] in an ·OH-induced cellular senescence model. Cyto·OH-generated lipid peroxide caused glutathione (GSH) and heme shortage and increased hydrogen peroxide (H_2_O_2_). Mechanistically, cyto·OH induced cellular senescence via the phosphorylation of ataxia telangiectasia mutated kinase serine 1981 (p-ATM^ser1981^)/p53 serine 15 (p-p53^ser15^)/p21 and phosphorylation of heme-regulated inhibitor (p-HRI)/phospho-eukaryotic translation initiation factor 2 subunit alpha serine 51 (p-eIF2α)/activating transcription factor 4 (ATF4)/p16 pathways. The authors suggest that H2 produced by gut bacteria has the potential to diffuse throughout the body to scavenge cyto ·OH in cells. Therefore, it is tempting to postulate that H_2_ produced by gut bacteria is involved in intracellular maintenance of the redox state, thereby suppressing cellular senescence and aging.

The mechanisms underlying the potential effects of collagen peptide (CP) in skin epidermal moisturization after ultraviolet B (UVB) irradiation were examined following the oral administration of CP in hairless mice [11]. The effects of CP were evaluated by measuring the transepidermal water loss (TEWL), skin hydration, wrinkle formation, and hyaluronic acid expression following exposure of skin to UVB irradiation. In this study, oral administration of CP led to increased mRNA and protein expression of hyaluronic acid synthases and a down-regulation of hyaluronidase (HYAL-1 and 2) mRNA expression concomitant with increased hyaluronic acid levels in skin tissue. In addition, the protein expression of skin-hydrating factors, filaggrin, and involucrin was upregulated. Altogether, the oral administration of CP seems to have a great potential to attenuate UVB-induced skin dehydration and wrinkle formation in vivo.

In conclusion, reports presented in this Special Issue add to the scientific evidence that nutrition plays a key role in the maintenance of healthy organs during aging. The food industry encounters many challenges and hurdles to provide functional and safe bioactive ingredients to support a healthy life and healthy aging [12]. Therefore, despite the enormous advances in recent years, additional well-designed and well-implemented basic and clinical research is necessary to unequivocally uncover the mechanisms of action and the true potential of nutritional intervention to delay senescence and aging in humans.
